# Editorial: Roles of metabolism, immunity, and microbiome in the pathophysiology of the breast cancer microenvironment

**DOI:** 10.3389/fonc.2026.2014425

**Published:** 2026-10-21

**Authors:** Saori Furuta

**Affiliations:** Case Western Reserve University School of Medicine, MetroHealth Medical Center Campus, Cleveland, OH, United States

**Keywords:** breast cancer;, cancer metabolism, gut microbiome, tumor immunology, tumor microenvironment

Breast cancer is a complex disease influenced by many factors beyond genetic changes in cancer cells. Metabolism, inflammation, immune responses, and the tumor microenvironment all affect breast cancer development, progression, and response to treatment. The overall health and metabolic condition of patients may also influence disease outcomes. The articles in this Research Topic examine these relationships, focusing on how metabolic and inflammatory processes within tumors and throughout the body may affect breast cancer.

One important topic is the relationship between systemic metabolism and breast cancer risk. In their original research article, *“Correlation study between inflammatory, nutritional and metabolic indicators and the risk of invasive ductal carcinoma of the breast,”* Chen et al. investigate how indicators of inflammation, nutrition, and metabolism are associated with the risk of invasive ductal carcinoma. These indicators can be measured using common clinical tests. Their findings suggest that metabolic and inflammatory conditions in the body may be related to breast cancer risk. This study highlights the importance of considering not only cancer cells but also the overall physiological condition of patients when studying breast cancer.

Changes in tumor metabolism may also affect the tumor microenvironment. In *“Lactylation-mediated remodelling of the breast cancer microenvironment: single-cell multidimensional analysis and prognostic model construction,”* Chen et al. examine the potential role of lactylation in breast cancer using single-cell and other molecular analyses. Lactylation is a process in which a lactyl group is added to a protein, potentially changing its function. Lactate was previously considered mainly a product of glucose metabolism. However, recent research suggests that lactate may also influence gene expression and cellular activity through lactylation. By analyzing different cell populations, this study explores how metabolic changes may affect cells in the tumor microenvironment. The findings also suggest that lactylation-related features may be useful for predicting clinical outcomes in breast cancer.

The location and distribution of cells within tumors are also important. In their review, *“Targeting the tripartite axis of immune-metabolic-spatial crosstalk to overcome therapy resistance in breast cancer,”* He et al. discuss how tumor metabolism, immune responses, and the spatial organization of cells interact in breast cancer. Different regions of a tumor may have different levels of oxygen and nutrients. Cancer cells and immune cells may also compete for these resources. In addition, the distribution of immune cells and surrounding stromal cells may affect their interactions. Together, these factors can influence the ability of immune cells to attack tumors and may contribute to resistance to cancer treatment. Understanding these interactions may help identify new strategies to improve treatment responses.

Metabolic hormones may provide another link between the body’s metabolic condition and breast cancer. In *“The role and research progress of FGF21 in breast cancer: a review,”* Chen et al. discuss the potential role of fibroblast growth factor 21 (FGF21) in breast cancer. FGF21 is a hormone involved in regulating glucose and lipid metabolism. Although its role in breast cancer is not yet fully understood, current evidence suggests that FGF21 signaling may influence cancer-related biological processes. Further research is needed to determine whether FGF21 could serve as a biomarker or therapeutic target. It is also important to understand whether its effects differ among breast cancer subtypes and patients with different metabolic conditions.

The gut microbiome may also influence breast cancer. In *“Beyond the tumor: the role of the gut microbiome in triple-negative breast cancer,”* Saadatkhah et al. review the relationship between intestinal microorganisms and triple-negative breast cancer. The gut microbiome can affect inflammation, immune responses, and the production of metabolites that influence cellular functions. These changes may affect tumor growth and response to treatment. However, further studies are needed to determine whether changes in the gut microbiome directly contribute to breast cancer progression or are associated with other disease-related factors. This research highlights the importance of studying the possible influence of the gut microbiome on breast cancer.

Metabolic changes may also contribute to symptoms experienced by patients with breast cancer. In *“Metabolomics-based exploration of the pathogenesis of breast cancer-related fatigue and progress of traditional Chinese medicine intervention,”* Jiang et al. discuss metabolic changes associated with breast cancer-related fatigue and possible approaches to managing this condition. Cancer-related fatigue is a common problem that can greatly affect patients’ daily activities and quality of life. Metabolomics, which measures small molecules involved in metabolism, may help identify biological changes associated with fatigue. Understanding these changes could help researchers identify biomarkers and develop better approaches to symptom management. This article highlights how metabolic research may benefit patients beyond improving tumor control.

Together, the six articles in this Research Topic demonstrate the importance of metabolism in several aspects of breast cancer. They address systemic inflammation and nutrition, lactylation in the tumor microenvironment, interactions between metabolism and immunity, hormonal regulation, the gut microbiome, and cancer-related fatigue (**Figure 1**). Although these studies focus on different aspects of the disease, they share an important message: breast cancer is influenced not only by cancer cells but also by their surrounding environment and the metabolic condition of the patient.

**Figure 1:**
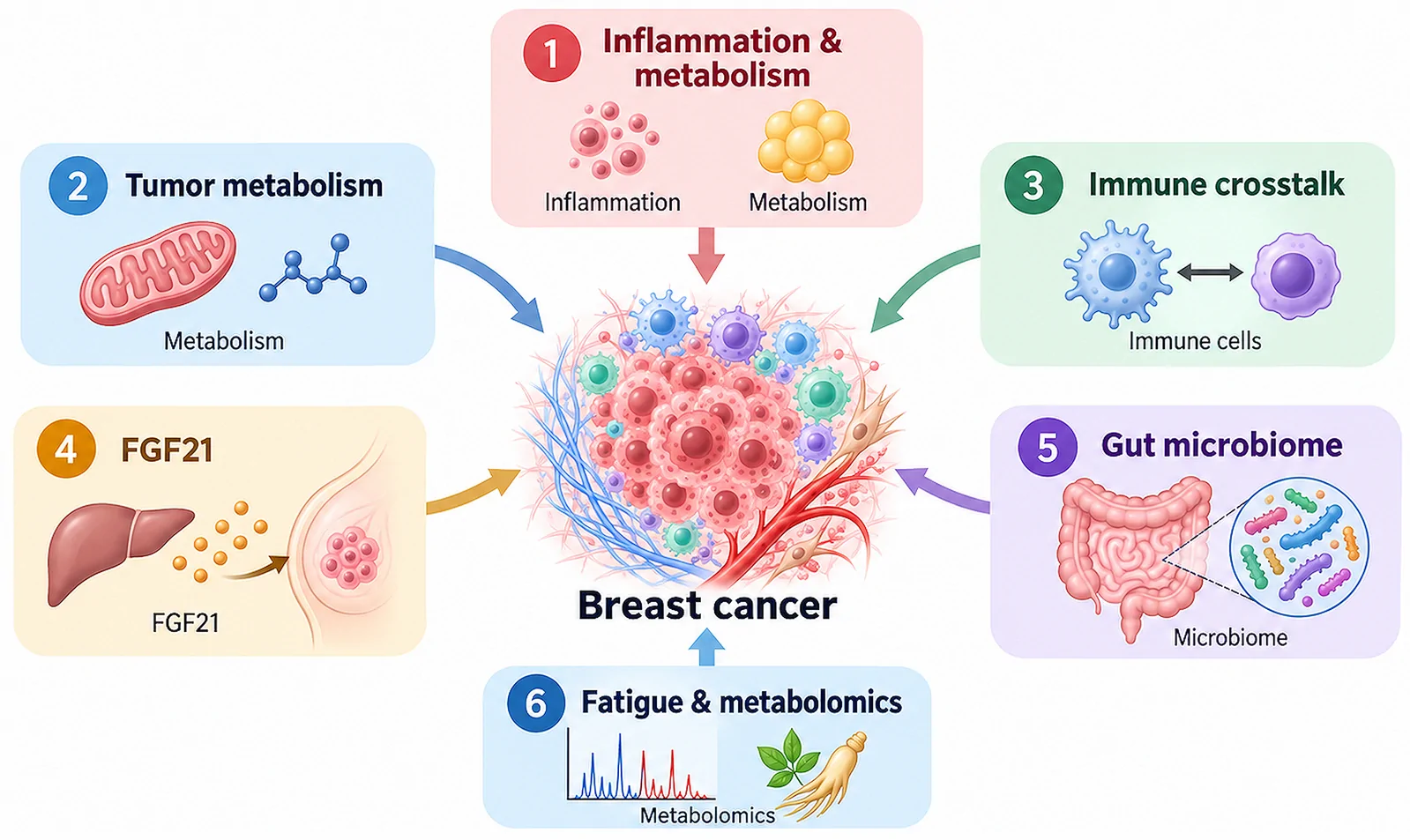
Metabolic and systemic factors associated with breast cancer. The image summarizes six topics covered in this Research Topic: inflammation and metabolism, tumor metabolism, immune interactions, FGF21 signaling, the gut microbiome, and cancer-related fatigue. Together, these factors may influence breast cancer biology, treatment response, and patient quality of life.

Several questions remain to be answered. Many findings from clinical observations, metabolomics, and single-cell analyses show associations, but additional experiments are needed to determine whether these factors directly affect breast cancer development or progression. Future studies should also examine whether these findings apply to different breast cancer subtypes, disease stages, and patient populations. Combining molecular analyses, immune profiling, microbiome research, and clinical data may help researchers better understand how these factors interact. Such studies could also distinguish metabolic changes that contribute to disease progression from changes that result from cancer or its treatment.

Overall, the articles in this Research Topic highlight the growing importance of understanding breast cancer metabolism in the context of the whole patient. Metabolism is not limited to cancer cells. It also affects immune cells, stromal cells, and other tissues throughout the body. By improving our understanding of these interactions, future research may identify new biomarkers, improve cancer treatments, and support better management of treatment-related symptoms. These advances could ultimately improve both treatment outcomes and quality of life for patients with breast cancer.

